# Mathematical Modelling of the Spatial Distribution of a COVID-19 Outbreak with Vaccination Using Diffusion Equation

**DOI:** 10.3390/pathogens12010088

**Published:** 2023-01-05

**Authors:** Brice Kammegne, Kayode Oshinubi, Oluwatosin Babasola, Olumuyiwa James Peter, Olumide Babatope Longe, Roseline Bosede Ogunrinde, Emmanuel Olurotimi Titiloye, Roseline Toyin Abah, Jacques Demongeot

**Affiliations:** 1 Department of Mathematics, University of Dschang, Dschang B.P. 96, Cameroon; 2 Laboratory AGEIS EA 7407, Team Tools for e-Gnosis Medical, Faculty of Medicine, University Grenoble Alpes (UGA), 38700 La Tronche, France; 3 Department of Mathematical Sciences, University of Bath, Bath BA2 7AY, UK; 4 Department of Mathematical and Computer Sciences, University of Medical Sciences, Ondo City PMB 536, Nigeria; 5 Department of Epidemiology and Biostatistics, School of Public Health, University of Medical Sciences, Ondo City PMB 536, Nigeria; 6 Faculty of Computational Sciences and Informatics, Academic City University, Accra AD 421, Ghana; 7 Department of Mathematics, Ekiti State University, Ado-Ekiti PMB 5363, Nigeria; 8 Department of Mathematics, University of Ilorin, Ilorin PMB 1515, Nigeria; 9 Department of Mathematics, University of Abuja, Abuja PMB 117, Nigeria

**Keywords:** COVID-19, SEIRV epidemic model, reaction–diffusion equation, basic reproduction number, vaccination, spatial distribution, infectious disease, pandemic, public health

## Abstract

The formulation of mathematical models using differential equations has become crucial in predicting the evolution of viral diseases in a population in order to take preventive and curative measures. In December 2019, a novel variety of Coronavirus (SARS-CoV-2) was identified in Wuhan, Hubei Province, China, which causes a severe and potentially fatal respiratory syndrome. Since then, it has been declared a pandemic by the World Health Organization and has spread around the globe. A reaction–diffusion system is a mathematical model that describes the evolution of a phenomenon subjected to two processes: a reaction process, in which different substances are transformed, and a diffusion process, which causes their distribution in space. This article provides a mathematical study of the Susceptible, Exposed, Infected, Recovered, and Vaccinated population model of the COVID-19 pandemic using the bias of reaction–diffusion equations. Both local and global asymptotic stability conditions for the equilibria were determined using a Lyapunov function, and the nature of the stability was determined using the Routh–Hurwitz criterion. Furthermore, we consider the conditions for the existence and uniqueness of the model solution and show the spatial distribution of the model compartments when the basic reproduction rate $R_{0}<1$ and $R_{0}>1$. Thereafter, we conducted a sensitivity analysis to determine the most sensitive parameters in the proposed model. We demonstrate the model’s effectiveness by performing numerical simulations and investigating the impact of vaccination, together with the significance of spatial distribution parameters in the spread of COVID-19. The findings indicate that reducing contact with an infected person and increasing the proportion of susceptible people who receive high-efficacy vaccination will lessen the burden of COVID-19 in the population. Therefore, we offer to the public health policymakers a better understanding of COVID-19 management.

## 1. Introduction

The use of differential equations, especially partial differential equations, has become very interesting to predict many evolutionary problems, in particular, the evolution of some biological phenomena. They are essential in fields such as aeronautical simulation, finance, weather prediction, and disease forecasting [1]. One of the most important classes of Partial Differential Equations is the class of reaction–diffusion equations. A reaction–diffusion system is a mathematical model that describes the evolution of a phenomenon subjected to two processes: a reaction process, in which different substances are transformed, and a diffusion process, which causes their distribution in space. Many research papers in epidemiology have proposed a modelling approach using real datasets from affected countries and have identified different characteristics controlled according to various parameters of the epidemic and to the effects of intervention strategies in the different countries concerned, depending on their situation. Ian Cooper and co-authors studied an SIR model of COVID-19 in diverse communities in Asia and North America [2]. In their study, they did not consider migration and death of individuals, so the population size remained constant during the study. In order to compensate for this deficiency, Ref. [3] conducted a study where the population size is not constant and the death rates of all compartments are the same. This led to more general results. Ref. [3] also studied the factors determining the spread of COVID-19 and used statistical modelling to propose strategies to prevent future accelerated viral infection similar to that observed in COVID-19. Furthermore, in [4], the authors presented a novel Susceptible–Infectious–Goneanewsusceptible–Recovered (SIGR) model to study the influence of vaccination at the sub-population level in the spread of COVID-19 pandemic, while in [5], authors proposed Susceptible–Exposed-Infectious–Recovered (SEIR) epidemic model with a convex incidence rate incorporated with a time delay in order to study the influence of delay in the dynamical system.

Other studies (such as [6,7,8,9,10,11,12]) have proposed spatial epidemic models. In these studies, reaction–diffusion equations were used to explain both the temporal and spatial evolution of the spread of diseases. Most of them use a continuous diffusion approach, but some others, such as Mimura’s team’s article, adopt the spatial SEIR model, in which individuals move randomly on a two-dimensional lattice with periodic boundary conditions [7]. Many authors used this class of equations to understand the behaviour of hepatitis C virus. The reaction term is the process of change of individuals involved in the interactions between species in the absence of diffusion, and the diffusion term describes the spatial movement of individuals [13,14,15,16]. In this paper, Ref. [17] studied a model of an SI diffusion reaction in which a pathogen is active in a population with two subgroups: healthy individuals who are susceptible to infection and already infected individuals who can transmit the disease to healthy individuals. To generalize this result to a heterogeneous population, Refs. [18,19] studied a reaction–diffusion Susceptible–Vaccinated–Infected–Recovered (SEIRV) model in a spatially heterogeneous environment with Dirichlet boundary conditions.

Furthermore, in [20], the authors developed a reaction–diffusion epidemic model on human mobility networks to characterize the spatio-temporal propagation of the COVID-19 pandemic, and a novel time-dependent function was incorporated into the model to describe the effects of human interventions. In [21], the authors studied an optimal control problem for a generalized multi-group reaction–diffusion SIR epidemic model, with heterogeneous nonlinear incidence rates, which is an extension of the study of an optimal control problem to a large class of reaction–diffusion multigroup epidemic models. In [22], the research carried out by the authors explored a novel SEIR-A reaction–diffusion COVID-19 epidemic system with direct and aerosol transmission. The effects of three strategies, including vaccination, receiving treatment, and wearing a mask, were evaluated numerically by authors. The findings of their research suggest that the three strategies can effectively control the peak and final scale of infection and shorten the duration of the COVID-19 epidemic.

The present work aims to carry out a mathematical study of a model of infection of COVID-19 by the bias of reaction–diffusion equations. In this current work, we want to make some additions to the work carried out earlier by researchers, by considering the exposed population (not still infectious) and the diffusion phenomena and by showing the existence, the uniqueness, the positiveness, and the boundedness of the solutions of the SEIRV diffusion reaction model considered. A study on the existence and nature of the equilibrium states is carried out according to the basic reproduction number $R_{0}$ whether it is less than or greater than 1. We were also able to find a Lyapunov function to investigate the global stability of the disease-free and endemic equilibria. For the confirmation of these properties, a numerical simulation is presented at the end of the study with Python software.

The remainder of the article is as follows: in Section 2, we present the mathematical model we formulated alongside parameters and their biological signification. In Section 3, an extensive mathematical analysis is presented to understand the dynamical behaviour of the system. In Section 4, we present a numerical simulation and sensitivity analysis of $R_{0}$ in order to identify parameters sensitive to the disease spread, and lastly in Section 5 and Section 6, we present some perspective, discussion and key conclusions derived from our research approach to the spatial distribution of COVID-19 outbreak with vaccination using diffusion equation.

## 2. Mathematical Model

**Figure 1:**
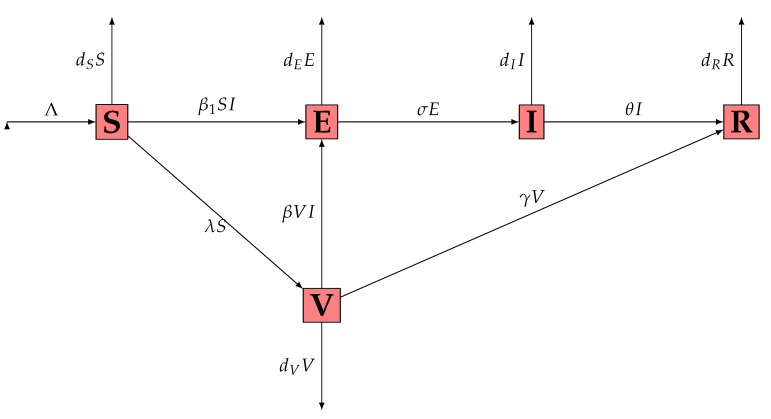
Interaction graph of the system (1).

Let us consider the following model:

(1) $$\left\{\begin{array}{ccc} \frac{\partial S(x,t)}{\partial t} & = & d_{1}\Delta S+\Lambda -d_{S}S-\beta_{1}SI-\lambda S,\\ \frac{\partial E(x,t)}{\partial t} & = & d_{2}\Delta E+\beta_{1}SI+\beta VI-\sigma E-d_{E}E\\ \frac{\partial I(x,t)}{\partial t} & = & d_{3}\Delta I+\sigma E-d_{I}I-\theta I,\\ \frac{\partial R(x,t)}{\partial t} & = & d_{4}\Delta R+\gamma V-d_{R}R+\theta I,\\ \frac{\partial V(x,t)}{\partial t} & = & d_{5}\Delta V+\lambda S-\beta VI-d_{V}V-\gamma V. \end{array}\right.$$

where *S*, *E*, *I*, *R*, and *V* are the population of susceptible, exposed, infected, recovered (and definitely immunized), and vaccinated at the position *x* at time *t*, respectively. The susceptible population reproduces at a constant rate Λ and is infected at a rate $\beta_{1}SI$, where $\beta_{1}$ is the contact rate per day between the susceptible and infected populations and $d_{S}$, $d_{E}$, $d_{I}$, $d_{R}$, and $d_{V}$ are, respectively, the mortality rates of susceptible, exposed, infected, recovered, and vaccinated people in the studied region. The parameter λ is the vaccination rate against COVID-19 in this population. The recovered population is produced from the infected at a rate θI. We assume that the population moves in the region Ω according to Fick’s law [1], and $d_{i}$’s being the diffusion coefficients and Δ the Laplacian operator. In this work, we consider the system (1) with initial conditions as follows

(2) $$S\left(x,0\right)=\varphi_{1}\left(x\right),E\left(x,0\right)=\varphi_{2}\left(x\right),I\left(x,0\right)=\varphi_{3}\left(x\right),V\left(x,0\right)=\varphi_{4}\left(x\right),R\left(x,0\right)=\varphi_{5}\left(x\right)\;x\in \Omega ,$$

where $\varphi_{i}\in C^{2}\left(\Omega \right)\cap C\left(\bar{\Omega }\right)$ and homogeneous Neumann boundary conditions

(3) $$\frac{\partial S}{\partial \nu }=\frac{\partial E}{\partial \nu }=\frac{\partial I}{\partial \nu }=\frac{\partial R}{\partial \nu }=\frac{\partial V}{\partial \nu }=0\;x\in \partial \Omega ,t>0,$$

where Ω is an open bounded subset of $R^{n}$ with smooth boundary ∂Ω, ν being the unit outer normal to ∂Ω. The interaction graph of the system (1) is presented in Figure 1 while the signification of parameters is presented in Table 1.

## 3. Dynamical Behaviour of the System and Qualitative Analysis

In this section, we study the dynamical behaviour of system (1), such as the existence and uniqueness of positive solutions and existence of equilibria, and their basic reproduction number, local stability, and global stability.

### 3.1. Existence, Uniqueness, and Positivity

**Definition 1.**  *Let $\left(\hat{S},\hat{E},\hat{I},\hat{V},\hat{R}\right)$ and $\left(\tilde{S},\tilde{E},\tilde{I},\tilde{V},\tilde{R}\right)$ in $C\left(\bar{\Omega }\times \left[0,\infty \right)\right)\cap C^{1,2}\left(\Omega \times \left[0,\infty \right)\right)$ are a pair of upper and lower solution to the problem (1), if $\tilde{S}\leq \hat{S}$, $\tilde{E}\leq \hat{E}$, $\tilde{I}\leq \hat{I}$, $\tilde{V}\leq \hat{V}$, $\tilde{R}\leq \hat{R}$ in $\bar{\Omega }\times \left[0,\infty \right)$, and the following differential inequalities hold:*

$$\begin{array}{lll} \frac{\partial \tilde{S}}{\partial t}\leq d_{1}\Delta \tilde{S}+\Lambda -\beta_{1}\tilde{S}\tilde{I}-\left(d_{S}+\lambda \right)\tilde{S} & ; & \frac{\partial \hat{S}}{\partial t}\geq d_{1}\Delta \hat{S}+\Lambda -\beta_{1}\hat{S}\hat{I}-\left(d_{S}+\lambda \right)\hat{S}\\ \frac{\partial \tilde{E}}{\partial t}\leq d_{2}\Delta \tilde{E}+\beta_{1}\tilde{S}\tilde{I}+\beta \tilde{V}\tilde{I}-\left(\sigma +d_{E}\right)\tilde{E} & ; & \frac{\partial \hat{E}}{\partial t}\geq d_{2}\Delta \hat{E}+\beta_{1}\hat{S}\hat{I}+\beta \hat{V}\hat{I}-\left(\sigma +d_{E}\right)\hat{E},\\ \frac{\partial \tilde{I}}{\partial t}\leq d_{3}\Delta \tilde{I}+\sigma \tilde{E}-(d_{1}+\theta )\tilde{I} & ; & \frac{\partial \hat{I}}{\partial t}\geq d_{3}\Delta \hat{I}+\sigma \hat{E}-(d_{1}+\theta )\hat{I},\\ \frac{\partial \tilde{R}}{\partial t}\leq d_{4}\sigma \tilde{R}+\gamma \tilde{I}-d_{R}\tilde{R}+\theta \tilde{I} & ; & \frac{\partial \hat{R}}{\partial t}\geq d_{4}\Delta \hat{R}+\gamma \hat{I}-d_{R}\hat{R}+\theta \hat{I},\\ \frac{\partial \tilde{V}}{\partial t}\leq d_{5}\Delta \tilde{R}+\lambda \tilde{S}-\beta \tilde{V}\tilde{I}-(d_{V}+\gamma )\tilde{V} & ; & \frac{\partial \hat{V}}{\partial t}\geq d_{5}\Delta \hat{R}+\lambda \hat{S}-\beta_{1}\hat{V}\hat{I}-(d_{V}+\gamma )\hat{V}. \end{array}$$

*for (x,t)∈Ω×(0,∞) and*

$$\begin{array}{llll} \frac{\partial \tilde{S}}{\partial \nu }\leq 0\leq \frac{\partial \hat{S}}{\partial \nu },\frac{\partial \tilde{E}}{\partial t}\leq 0\leq \frac{\partial \hat{E}}{\partial \nu }, & \frac{\partial \tilde{I}}{\partial \nu }\leq 0\leq \frac{\partial \hat{I}}{\partial \nu },\frac{\partial \tilde{V}}{\partial \nu }\leq 0\leq \frac{\partial \hat{V}}{\partial t}, & \frac{\partial \tilde{R}}{\partial \nu }\leq 0\leq \frac{\partial \hat{R}}{\partial \nu },\;\mathrm{for}\;\left(x,t\right)\in \partial \Omega \times \left(0,\infty \right) & \\ \tilde{S}\left(x,t\right)\leq \varphi_{1}\left(x,t\right)\leq \hat{S}\left(x,t\right), & \tilde{E}\left(x,t\right)\leq \varphi_{2}\left(x,t\right)\leq \hat{E}\left(x,t\right), & \tilde{I}\left(x,t\right)\leq \varphi_{3}\left(x,t\right)\leq \hat{I}\left(x,t\right),\\ \tilde{V}\left(x,t\right)\leq \varphi_{4}\left(x,t\right)\leq \hat{V}\left(x,t\right), & \tilde{R}\left(x,t\right)\leq \varphi_{5}\left(x,t\right)\leq \hat{R}\left(x,t\right), & \mathrm{for}\;\left(x,t\right)\in \partial \Omega \times \left(0,\infty \right) \end{array}$$

It is easy to see that $\underline{0}$ =(0,0,0,0,0) and K $=(K_{1},K_{2},K_{3},K_{4},K_{5})$ are a pair of coupled lower-upper solutions to problem (1), where

$$\begin{array}{ccc} K_{1} & = & \max\{\frac{\Lambda }{d},∥\varphi_{1}{∥}_{C(\bar{\Omega },R)}\}\\ K_{2} & = & \max\{\frac{\Lambda }{d},∥\varphi_{2}{∥}_{C(\bar{\Omega },R)}\}\\ K_{3} & = & \max\{\frac{\sigma \Lambda }{d^{2}},∥\varphi_{3}{∥}_{C(\bar{\Omega },R)}\}\\ K_{4} & = & \max\{\frac{d\gamma \Lambda +\gamma \Lambda \theta }{d^{3}},∥\varphi_{4}{∥}_{C(\bar{\Omega },R)}\}\\ K_{5} & = & \max\{\frac{\lambda \Lambda }{d^{2}},∥\varphi_{5}{∥}_{C(\bar{\Omega },R)}\}, \end{array}$$

and $d=\min\{d_{S}+\lambda ;\sigma +d_{E};d_{I}+\theta ;d_{R}\}$. Using the following lemma provided by Redinger [13], we obtain the existence and uniqueness of the solution.

**Lemma 1.**  *Let $\hat{U}$ and $\tilde{U}$ be a pair of upper and lower solutions for problem (1) and suppose that the initial functions $\varphi_{i}$(i=1,2…,5) are continuous in $\bar{\Omega }$. Then problem (1) has exactly one regular solution U(x,t)=(S(x,t),E(x,t),I(x,t),V(x,t),R(x,t)) satisfying $\tilde{U}\leq U\leq \hat{U}$ in $\bar{\Omega }\times \left[0,\infty \right)$.*

Hence, $0\leq S\left(x,t\right)\leq K_{1}$, $0\leq E\left(x,t\right)\leq K_{2}$, $0\leq I\left(x,t\right)\leq K_{3}$, $0\leq V\left(x,t\right)\leq K_{4}$, $0\leq R\left(x,t\right)\leq K_{5}$. Furthermore, also, by the maximum principle, if $\varphi_{i}\left(x\right)\neq 0$, we have S(x,t)>0,E(x,t)>0,I(x,t)>0,V(x,t)>0,R(x,t)>0 for all $t>0,x\in \bar{\Omega }$.

### 3.2. Equilibria and Basic Reproduction Number

#### 3.2.1. Disease-Free Equilibrium and Basic Reproduction Number

It is easy to verify that system (1) always has a disease-free equilibrium given by $\left(S_{0},0,0,0,V_{0}\right)$ where

$$\begin{array}{ccc} S_{0} & = & \frac{\Lambda }{d_{S}+\lambda }\\ V_{0} & = & \frac{\lambda }{d_{V}+\gamma }\frac{\Lambda }{d_{S}+\lambda }. \end{array}$$

In order to find the basic reproduction number $R_{0}$ of the system (1), we obtain the following linear system at disease-free equilibrium $E_{0}$:

(4) $$\left\{\begin{array}{cc} \frac{\partial E(x,t)}{\partial t}=d_{2}\Delta E+\beta_{1}SI+\beta VI-\sigma E-d_{E}E, & x\in \Omega ,t>0\\ \frac{\partial I(x,t)}{\partial t}=d_{3}\Delta I+\sigma E-d_{I}I-\theta I, & x\in \Omega ,t>0\\ \frac{\partial R(x,t)}{\partial t}=d_{4}\Delta R+\gamma V-d_{R}R+\theta I, & x\in \Omega ,t>0\\ \frac{\partial E}{\partial \nu }=\frac{\partial I}{\partial \nu }=\frac{\partial R}{\partial \nu }=0 & x\in \partial \Omega ,t>0. \end{array}\right.$$

By following the idea of Wendi Wang [23], consider the vectors F and V given by

$$F=\left(\begin{array}{c} \beta_{1}SI+\beta \;VI\\ \sigma \;E\\ \gamma V+\theta I \end{array}\right),\;V=\left(\begin{array}{c} \sigma E+d_{E}\;E\\ d_{I}I+\theta \;I\\ d_{R}R \end{array}\right).$$

where F is the input rate vector and V the transfer rate vector for each compartment. In order to find the next generation matrix K, we have

$$JF\left(E_{0}\right)=\left(\begin{array}{ccc} 0 & \frac{\Lambda }{d_{S}+\lambda }\left(\beta_{1}+\frac{\beta \lambda }{d_{V}+\gamma }\right) & 0\\ \sigma & 0 & 0\\ 0 & \theta & 0 \end{array}\right),\;JV\left(E_{0}\right)=\left(\begin{array}{ccc} \sigma +d_{E} & 0\\ 0 & d_{I}+\theta & 0\\ 0 & 0 & d_{R} \end{array}\right).$$

Therefore, K is given by

$$K=JF\left(E_{0}\right)JV^{-1}\left(E_{0}\right)=\left(\begin{array}{ccc} 0 & \frac{\beta_{1}S_{0}+\beta V_{0}}{d_{I}+\theta } & 0\\ \frac{\sigma }{\sigma +d_{E}} & 0 & 0\\ 0 & 0 & 0 \end{array}\right).$$

Finally, the basic reproduction number is given by

(5) $$R_{0}=\rho \left(K\right)=\frac{\sigma \Lambda (\beta_{1}\left(d_{V}+\gamma \right)+\beta \lambda )}{\left(d_{S}+\lambda \right)\left(d_{V}+\gamma \right)\left(\sigma +d_{E}\right)\left(d_{I}+\theta \right)}.$$

#### 3.2.2. Existence and Uniqueness of the Endemic Equilibrium

For the second equilibrium, we are looking for $E^{*}=\left(S^{*},E^{*},I^{*},R^{*},V^{*}\right)$ all different from 0 such that

(6) $$\begin{array}{ccc} \Lambda -d_{S}S-\beta_{1}SI-\lambda S & = & 0\\ \beta_{1}SI+\beta VI-\sigma E-d_{E}E & = & 0\\ \sigma E-(d_{I}+\theta )I & = & 0\\ \gamma V-d_{R}R+\theta I & = & 0\\ \lambda S-\beta VI-d_{V}V-\gamma V & = & 0. \end{array}$$

From these equations, we come out with

(7) $$\begin{array}{ccc} S=f_{S}\left(I\right) & = & \frac{\Lambda }{d_{S}+\lambda +\beta_{1}I}\\ E=f_{E}\left(I\right) & = & \frac{I(d_{I}+\theta )}{\sigma }\\ V=f_{V}\left(I\right) & = & \frac{\lambda f_{S}\left(I\right)}{\beta I+d_{V}+\gamma }\\ R=f_{R}\left(I\right) & = & \frac{\gamma f_{V}\left(I\right)+\theta I}{d_{R}}. \end{array}$$

By using (6) and (7), we will prove the existence of solution of equation

(8) $$G\left(I\right)=\beta_{1}If_{S}\left(I\right)+\beta If_{V}\left(I\right)-\left(\sigma +d_{E}\right)f_{E}\left(I\right)=0.$$

It is easy to see that G(0)=0 and $\lim_{I\to +\infty }G\left(I\right)=-\infty$. Therefore, the previous equation has a solution if $G^{\prime }\left(0\right)>0$. However, we have

(9) $$\begin{array}{ccc} G^{\prime }\left(0\right) & = & \beta_{1}f_{S}\left(0\right)+\beta h\left(0\right)-\left(\sigma +d_{E}\right)g^{\prime }\left(0\right) \end{array}$$

(10) $$\begin{array}{ccc} & = & \frac{\beta_{1}\Lambda }{d_{S}+\lambda }+\frac{\beta \lambda \Lambda }{\left(d_{V}+\gamma \right)\left(d_{S}+\lambda \right)}-\frac{\left(\sigma +d_{E}\right)\left(d_{I}+\theta \right)}{\sigma }. \end{array}$$

It follows that $G^{\prime }\left(0\right)>0$ if and only if

$$0<\frac{\beta_{1}\Lambda }{d_{S}+\lambda }+\frac{\beta \lambda \Lambda }{\left(d_{V}+\gamma \right)\left(d_{S}+\lambda \right)}-\frac{\left(\sigma +d_{E}\right)\left(d_{I}+\theta \right)}{\sigma }.$$

This means

$$\begin{array}{ccc} \frac{\left(\sigma +d_{E}\right)\left(d_{I}+\theta \right)}{\sigma } & < & \frac{\beta_{1}\Lambda }{d_{S}+\lambda }+\frac{\beta \lambda \Lambda }{\left(d_{V}+\gamma \right)\left(d_{S}+\lambda \right)}\\ 1 & < & \frac{\Lambda \sigma \left(\beta_{1}\left(d_{V}+\gamma \right)+\beta \lambda \right)}{\left(\sigma +d_{E}\right)\left(d_{I}+\theta \right)\left(d_{S}+\lambda \right)\left(d_{V}+\gamma \right)}\equiv R_{0}. \end{array}$$

To prove uniqueness, we are looking for a solution I≠0 which verifies (8). Since I≠0, it can divided through, and then using (7), we obtain

$$h\left(I\right)=\frac{\beta_{1}\Lambda }{d_{S}+\lambda +\beta_{1}I}+\frac{\beta \lambda \Lambda }{\left(d_{S}+\lambda +\beta_{1}I\right)\left(\beta I+d_{V}+\gamma \right)}-\frac{\left(\sigma +d_{E}\right)\left(d_{I}+\theta \right)}{\sigma },$$

and it follows that

(11) $$h^{\prime }\left(I\right)=-\frac{\beta_{1}^{2}\Lambda }{{\left(d_{S}+\lambda +\beta_{1}I\right)}^{2}}-\frac{\beta \Lambda \lambda \left(\beta_{1}\left(\beta I+d_{V}+\gamma \right)+\beta \left(d_{S}+\lambda +\beta_{1}I\right)\right)}{{\left(d_{S}+\lambda +\beta_{1}I\right)}^{2}{\left(\beta I+d_{V}+\gamma \right)}^{2}}<0.$$

Finally, the function *G* is injective. Therefore, if $I_{1}$ and $I_{2}$ are two solutions of (8), then $G\left(I_{1}\right)=G\left(I_{2}\right)=0$ and by injectivity of *G*, we have $I_{1}=I_{2}$. This allows us to conclude the uniqueness of the endemic equilibrium. We come out with this following theorem.

**Theorem 1.**  If $R_{0}>1$, then the infected equilibrium $E^{*}=\left(S^{*},E^{*},I^{*},R^{*},V^{*}\right)$ exists and is unique.

### 3.3. Stability of Equilibrium

Let $0=\mu_{0}<\mu_{i}<\mu_{i+1}$ for i=1,2,… be the eigenvalues of −Δ on Ω with homogeneous Neumann boundary conditions, $E(\mu_{i})$ is the space of eigenfunctions corresponding to $\mu_{i}$’s and $\left\{\phi_{ij}:j=1,2,\ldots ,\dim E\left(\mu_{i}\right)\right\}$ an orthogonal basis of $E(\mu_{i})$. Then, $X={\left[C^{1}\left(\bar{\Omega }\right)\right]}^{3}$ can be decomposed as

$$X=\overset{\infty }{\underset{i=1}{⨁}}X_{i},\;X_{i}=\overset{\dim E(\mu_{i})}{\underset{i=1}{⨁}}X_{ij}$$

where $X_{ij}=\left\{c\phi_{ij}:c\in R^{3}\right\}$. Then we can prove the local stability of equilibrium as in [2,24].

**Theorem 2.**  *If $R_{0}<1$, then the disease-free equilibrium $E_{0}$ of system (1) is locally asymptotically stable.*

**Proof.**  The linearisation of system (1) at $E_{0}$ can be expressed by

$$\frac{\partial Z(x,t)}{\partial t}=D\Delta Z\left(x,t\right)+AZ\left(x,t\right),$$

where Δ is the Jacobian matrix of system (1) and A is the residual non-linear operator Z=(S,E,I,R,V), $D=diag(d_{1},d_{2},d_{3},d_{4},d_{5})$, and if J is the Jacobian matrix of system (1), we have:

(12) $$-J+D\Delta =\left(\begin{array}{ccccc} -(d_{S}+\lambda )-\beta I & 0 & -\beta_{1}S & 0 & 0\\ \beta_{I} & -(\sigma +d_{E}) & \beta_{1}S+\beta V & 0 & \beta I\\ 0 & \sigma & -(d_{I}+\theta ) & 0 & 0\\ 0 & 0 & \theta & -d_{R} & \gamma \\ \lambda & 0 & -\beta V & 0 & -\beta I-(d_{I}+\gamma ). \end{array}\right)$$

Therefore, the characteristic equation of −J at $E_{0}$ is

(13) $$\left(-d_{R}-\mu_{i}d_{4}-x\right)\left(-\left(d_{S}+\lambda \right)-\mu_{i}d_{1}-x\right)\left(-\left(d_{I}+\gamma \right)-\mu_{i}d_{3}-x\right)P\left(x\right),$$

where

$$P\left(x\right)=x^{2}+\left(\sigma +d_{E}+d_{I}+\theta +\mu_{i}\left(d_{2}+d_{5}\right)\right)x+\left(\sigma +d_{E}\right)\left(d_{I}+\theta \right)-\sigma \left(\beta_{1}S_{0}+\beta V_{0}\right).$$

It is obvious that (13) has as eigenvalues

$$x_{1}=-d_{R}-\mu_{i}d_{4},\;x_{2}=-\left(d_{S}+\lambda \right)-\mu_{i}d_{1},\;x_{3}=-\left(d_{I}+\gamma \right)-\mu_{i}d_{3}.$$

The two other ones are $x_{4}$ and $x_{5}$ such that

$$\begin{array}{ccc} x_{4}+x_{5} & = & -(\sigma +d_{E}+d_{I}+\theta +\mu_{i}\left(d_{2}+d_{5}\right))\\ x_{4}x_{5} & = & \left(\sigma +d_{E}\right)\left(d_{I}+\theta \right)-\sigma \left(\beta_{1}S_{0}+\beta V_{0}\right). \end{array}$$

They are negative if and only if $x_{4}x_{5}>0$; that is,

(14) $$0<\left(\sigma +d_{E}\right)\left(d_{I}+\theta \right)-\sigma \left(\beta_{1}S_{0}+\beta V_{0}\right).$$

By using the values of $S_{0}$ and $V_{0}$ given by (6), Equation (14) is equivalent to

$$\sigma \left(\beta_{1}S_{0}+\beta V_{0}\right)<\left(\sigma +d_{E}\right)\left(d_{I}+\theta \right).$$

This implies

$$\frac{\sigma \Lambda }{d_{S}+\lambda }\left(\frac{\beta \lambda }{d_{V}+\gamma }+\beta_{1}\right)<\left(\sigma +d_{E}\right)\left(d_{I}+\theta \right),$$

and it follows that $\frac{\sigma \Lambda }{d_{S}+\lambda }\frac{\beta_{1}\left(d_{V}+\gamma \right)+\beta \lambda }{d_{V}+\gamma }<\left(\sigma +d_{E}\right)\left(d_{I}+\theta \right)$. Finally, we come out with

$$R_{0}\equiv \frac{\sigma \Lambda (\beta_{1}\left(d_{V}+\gamma \right)+\beta \lambda )}{\left(d_{S}+\lambda \right)\left(d_{V}+\gamma \right)\left(\sigma +d_{E}\right)\left(d_{I}+\theta \right)}<1,$$

and the result follows. □

**Theorem 3.**  If $R_{0}>1$, then the endemic equilibrium $E^{*}$ of system (1) is locally asymptotically stable.

**Proof.**  The linearization of system (1) at $E^{*}$ can be expressed by

$$\frac{\partial Z(x,t)}{\partial t}=D\Delta Z\left(x,t\right)+BZ\left(x,t\right),$$

where

$$-J+D\Delta =\left(\begin{array}{ccccc} -\left(d_{S}+\lambda +\beta_{1}I^{*}\right) & 0 & -\beta_{1}S^{*} & 0 & 0\\ \beta_{1}I^{*} & -(\sigma +d_{E}) & \beta_{1}S^{*}+\beta V^{*} & 0 & \beta I^{*}\\ 0 & \sigma & -(d_{I}+\theta ) & 0 & 0\\ 0 & 0 & \theta & -d_{R} & \gamma \\ \lambda & 0 & -\beta V^{*} & 0 & -\left(\beta I^{*}+d_{I}^{*}+\gamma \right) \end{array}\right),$$

and $D=(d_{1},d_{2},d_{3},d_{4},d_{5})$.

Thus, the characteristic equation at $E^{*}$ is

(15) $$\left(-d_{R}-\mu_{i}d_{4}-x\right)\left(x^{4}+\lambda_{3}x^{3}+\lambda_{2}x^{2}+\lambda_{1}x+\lambda_{0}\right)=0,$$

where

$$\begin{array}{ccc} \lambda_{3} & = & (\left(d_{S}+\lambda +\beta_{1}I^{*}\right)+\mu_{i}d_{1}+\left(\beta I^{*}+d_{V}+\gamma \right)+\mu_{i}d_{5}+\left(\sigma +d_{E}\right)+\mu_{i}d_{2}+\left(d_{I}+\theta \right)+\mu_{i}d_{3})>0\\ \lambda_{2} & = & -\left(d_{S}+\lambda +\beta_{1}I^{*}\right)+\mu_{i}d_{1}\left(\beta I^{*}+d_{V}+\gamma +\mu_{i}d_{5}\right)\sigma \beta_{1}S^{*}+\beta V^{*}\left(\sigma +d_{E}+\mu_{i}d_{2}\right)\left(d_{I}+\theta +\mu_{i}d_{3}\right)>0\\ \lambda_{1} & = & \left(\sigma \beta_{1}S^{*}+\beta V^{*}\right)\left(1+\left(d_{S}+\lambda +\beta_{1}I^{*}\right)+\mu_{i}d_{1}+\left(\beta I^{*}+d_{V}+\gamma \right)+\mu_{i}d_{5}\right)+\beta \beta_{1}\sigma I^{*}\left(S^{*}-V^{*}\right)\\ \lambda_{0} & = & \left(d_{S}+\lambda +\beta_{1}I^{*}+\mu_{i}d_{1}\right)\left(\beta I^{*}+d_{V}+\gamma +\mu_{i}d_{5}\right)\left(\sigma +d_{E}+\mu_{i}d_{2}\right)\left(d_{I}+\theta +\mu_{i}d_{3}\right)\\ \; & \; & -\beta \beta_{1}\sigma I^{*}\left(\lambda S^{*}-\left(\beta I^{*}+d_{V}+\gamma +\mu_{i}d_{5}\right)S^{*}-V^{*}\left(d_{S}+\lambda +\beta_{1}I^{*}+\mu_{i}d_{1}\right)+\right)\\ \; & \; & -\sigma \left(\beta I^{*}+d_{V}+\gamma +\mu_{i}d_{5}\right)\beta_{1}S^{*}+\beta V^{*}\left(A+1\right). \end{array}$$

Hence, $\lambda_{0},\lambda_{2},\lambda_{3}>0$ and $\lambda_{1}\left(\lambda_{3}\lambda_{2}-\lambda_{1}\right)>\lambda_{3}^{2}\lambda_{0}$ whenever $R_{0}>1$. Then, using the Routh–Hurwitz criterion, we claim that all eigenvalues of (15) have negative real parts. Thus, the endemic equilibrium $E^{*}$ of system (1) is locally asymptotically stable when $R_{0}>1$. This completes the proof. □

### 3.4. Global Stability of the Disease-Free and Endemic Equilibria by Means of a Lyapunov Function

In this section, we investigate the global stability of the disease-free equilibrium $E_{0}$ for system (1). We consider a Lyapunov functional based on the Volterra function Φ(x)=x−1−lnx. Clearly, Φ(x)≥0 for all x>0 and the equality holds if and only if x=1. In the presence of diffusion, the aim is to show that every solution of system (1) with a positive initial value that is different from the equilibrium point will converge to the equilibrium.

**Theorem 4.**  *If $R_{0}<1$, then the disease-free equilibrium $E_{0}$ of system (1) is globally asymptotically stable in the feasible region *Γ*. If $R_{0}>1$, then $E_{0}$ is unstable.*

**Proof.**  Define a Lyapunov function

$$L\left(t\right)=\int_{\Omega }L_{1}\left(x,t\right)dx,$$

where

$$L_{1}\left(x,t\right)=aE\left(x,t\right)+bI\left(x,t\right)$$

and a,b are positive constants to be determined later. Then, along with the solutions of system (1), we have

$$\begin{array}{ccc} \frac{\partial L_{1}}{\partial t} & = & a\frac{\partial E}{\partial t}+b\frac{\partial I}{\partial t}\\ \; & = & a\left(\beta_{1}SI+\beta VI-\sigma E-d_{E}E\right)+b\left(\sigma E-\left(d_{I}+\theta \right)I\right)+ad_{2}\Delta E+bd_{3}\Delta \;I\\ \; & \leq & a\left(\beta_{1}S_{0}I+\beta V_{0}I-\sigma E-d_{E}E\right)+b\left(\sigma E-\left(d_{I}+\theta \right)I\right)+ad_{2}\Delta E+bd_{3}\Delta \;I\\ \; & = & I\left(a\beta_{1}S_{0}+a\beta V_{0}-b\left(d_{I}+\theta \right)\right)+\left(b\sigma -a(\sigma +d_{E})\right)E+ad_{2}\Delta E+bd_{3}\Delta I \end{array}$$

By choosing a=σ and $b=\sigma +d_{E}$, we obtain

$$\frac{\partial L_{1}}{\partial t}\leq \left(d_{I}+\theta \right)\left(\sigma +d_{E}\right)\left(R_{0}-1\right)I+ad_{2}\Delta E+bd_{3}\Delta I.$$

Using Green’s formula and the Neumann boundary conditions in (3), we obtain

$$\int_{\Omega }\Delta Idx=\int_{\Omega }\Delta Edx=0.$$

Using the above conditions, we have

(16) $$\frac{\partial L}{\partial t}\leq \int_{\Omega }\left(\left(d_{I}+\theta \right)\left(\sigma +d_{E}\right)\left(R_{0}-1\right)I\right)dx.$$

Therefore, $\frac{\partial L}{\partial t}\leq 0$ whenever $R_{0}<1$. Furthermore, $\frac{\partial L}{\partial t}=0$ if and only if I=0. It follows that the largest invariant subset $\left\{\left(S,E,I,V,R\right)\;\mathrm{such}\;\mathrm{that}\;\dot{L}=0\right\}$ when $R_{0}<1$ is reduced to the singleton $E_{0}$. By LaSalle’s Invariance Principle [25], the infection-free equilibrium of system (1) is globally asymptotically stable when $R_{0}<1$ and if $R_{0}>1$, then $E_{0}$ is unstable. □

**Theorem 5.**  *Consider a Lyapunov function*

$$H\left(t\right)=\int_{\Omega }H_{1}\left(x,t\right)dx,$$

*with*

$$H_{1}\left(x,t\right)=S^{*}\Phi \left(\frac{S}{S^{*}}\right)+I^{*}\Phi \left(\frac{I}{I^{*}}\right)+R^{*}\Phi \left(\frac{R}{R^{*}}\right).$$

*where*

Φ(x)=x−1−lnx

Then, H is non-negative and is strictly minimized at the unique equilibrium $\left(S^{*},E^{*},I^{*},R^{*},V^{*}\right)$; i.e., it is a valid Lyapunov function. Hence, $E^{*}=\left(S^{*},E^{*},I^{*},R^{*},V^{*}\right)$ is globally asymptotically stable.

**Proof.**  According to (1), we have

$$\begin{array}{ccc} \frac{\partial H(x,t)}{\partial t} & = & \left(1-\frac{S^{*}}{S}\right)\frac{\partial S}{\partial t}+\left(1-\frac{I^{*}}{I}\right)\frac{\partial I}{\partial t}+\left(1-\frac{R^{*}}{R}\right)\frac{\partial R}{\partial t}\\ \; & = & \left(1-\frac{S^{*}}{S}\right)\left(d_{1}\Delta S+\Lambda -d_{S}S-\beta_{1}SI-\lambda S\right)+\left(1-\frac{I^{*}}{I}\right)\left(d_{3}\Delta I+\sigma E-d_{I}I-\theta I\right)\\ \; & + & \left(1-\frac{R^{*}}{R}\right)\left(d_{4}\Delta R+\gamma V-d_{R}R+\theta I\right)\\ \; & = & -\frac{{\left(S^{*}-S\right)}^{2}\left(d_{S}+\lambda \right)}{S}+\left(1-\frac{S^{*}}{S}\right)d_{1}\Delta S+\beta_{1}S^{*}I^{*}\left(1-\frac{SI}{S^{*}I^{*}}-\frac{S^{*}}{S}+\frac{I}{I^{*}}\right)\\ \; & + & \sigma \frac{\left(I-I^{*}\right)\left(E-E^{*}\right)}{I}-\frac{\left(d_{I}+\theta \right){\left(I-I^{*}\right)}^{2}}{I}+\left(1-\frac{I^{*}}{I}\right)d_{3}\Delta I+\left(1-\frac{R^{*}}{R}\right)\frac{\partial R}{\partial t}\\ \; & = & -\frac{{\left(S^{*}-S\right)}^{2}\left(d_{S}+\lambda \right)}{S}+\left(1-\frac{S^{*}}{S}\right)d_{1}\Delta S-\beta_{1}S^{*}I^{*}\left(\Phi \left(\frac{SI}{S^{*}I^{*}}\right)+\Phi \left(\frac{S^{*}}{S}\right)-\Phi \left(\frac{I}{I^{*}}\right)\right)\\ \; & + & \left(1-\frac{I^{*}}{I}\right)d_{3}\Delta I-\sigma E^{*}I^{*}\left(\Phi \left(\frac{I}{I^{*}}\right)+\Phi \left(\frac{E}{E^{*}}\right)-\Phi \left(\frac{EI}{E^{*}I^{*}}\right)\right)\\ \; & - & \frac{d_{R}}{R}{\left(R^{*}-R\right)}^{2}-R^{*}I^{*}\left(\Phi \left(\frac{I}{I^{*}}\right)+\Phi \left(\frac{R}{R^{*}}\right)-\Phi \left(\frac{RI}{R^{*}I^{*}}\right)\right)+\left(1-\frac{R^{*}}{R}\right)d_{4}\Delta R. \end{array}$$

Using Green’s formula and the Neumann boundary conditions in (3), we obtain

$$\int_{\Omega }\left(1-\frac{S^{*}}{S}\right)d_{1}\Delta Sdx=-d_{1}\int_{\Omega }\nabla \left(1-\frac{S^{*}}{S}\right)\nabla Sdx=-d_{1}\int_{\Omega }\frac{S^{*}}{S^{2}}{\left|\nabla S\right|}^{2}dx\leq 0,$$

$$\int_{\Omega }\left(1-\frac{V^{*}}{V}\right)d_{1}\Delta Vdx=-d_{1}\int_{\Omega }\nabla \left(1-\frac{V^{*}}{V}\right)\nabla Vdx=-d_{1}\int_{\Omega }\frac{V^{*}}{V^{2}}{\left|\nabla V\right|}^{2}dx\leq 0,$$

$$\int_{\Omega }\left(1-\frac{R^{*}}{R}\right)d_{1}\Delta Rdx=-d_{1}\int_{\Omega }\nabla \left(1-\frac{R^{*}}{R}\right)\nabla Rdx=-d_{1}\int_{\Omega }\frac{R^{*}}{R^{2}}{\left|\nabla R\right|}^{2}dx\leq 0.$$

Using the above conditions, we conclude that

(17) $$\frac{dH(t)}{dt}\leq \int_{\Omega }\left[-\frac{{\left(S^{*}-S\right)}^{2}\left(d_{S}+\lambda \right)}{S}-\frac{d_{R}}{R}{\left(R^{*}-R\right)}^{2}-\frac{\left(d_{I}+\theta \right){\left(I-I^{*}\right)}^{2}}{I}\right].$$

Furthermore, we have $\frac{dH(t)}{dt}=0$ only at steady-state $E^{*}=\left(S^{*},E^{*},I^{*},R^{*},V^{*}\right)$. Therefore, by Lyapunov’s direct method, the steady state solution $E^{*}$ is globally asymptotically stable. □

## 4. Numerical Simulation and Sensitivity Analysis of $R_{0}$

### 4.1. Numerical Simulation

#### 4.1.1. Experiment 1: Numerical Simulation When $R_{0}<1$

Here, n=1 (spatial dimension). We choose

(18) $$\left(d_{1},d_{2},d_{3},d_{4},d_{5}\right)=\left(1000,900,20,900,1200\right)m^{2}{\mathrm{day}}^{-1}.$$

**Figure 2:**
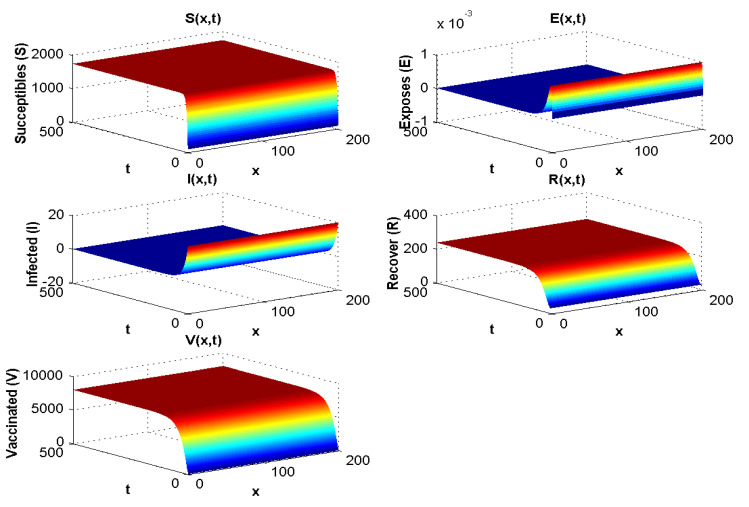
Spatial and temporal distribution when $R_{0}<1$.

With the values in Table 2 and simulation, we obtain $R_{0}=2.94^{-6}<1$ and at the disease-free equilibrium, we show $S_{0}$ to be 1800 and $V_{0}$ to be 8000. Figure 2 displays the solution curves of the model where they tend to the stability of the disease-free equilibrium point with different initial histories. Hence, numerical simulations of Experiment 1 confirm the qualitative results.

#### 4.1.2. Experiment 2: Numerical Simulation When $R_{0}>1$

Here, n=1 (spatial dimension), and we use the same diffusion coefficient as presented in experiment 1.

**Figure 3:**
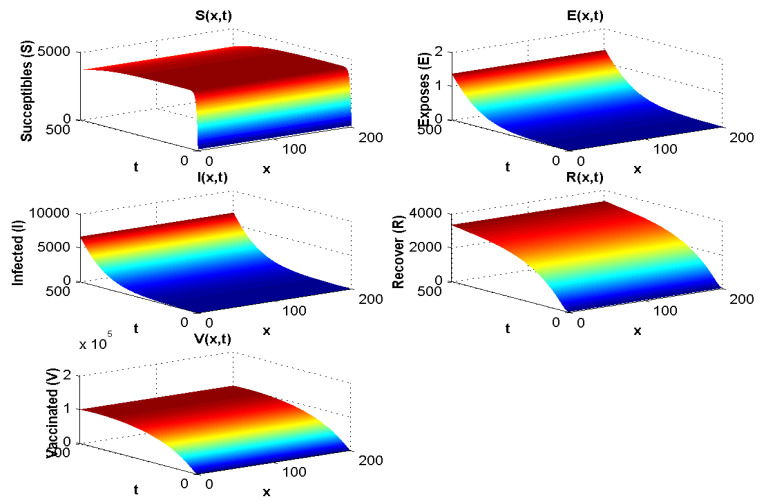
Spatial and temporal distribution when $R_{0}>1$.

With the values in Table 3, the endemic equilibrium value is $E^{*}=(4000,1.25,6000,$$3500,10^{5})$, and $R_{0}=2.05>1$. The result is presented in Figure 3 and is consistent with the stability of endemic equilibrium presented in the qualitative analysis.

#### 4.1.3. Experiment 3: Numerical Simulation When $R_{0}<1$ and λ=0

**Figure 4:**
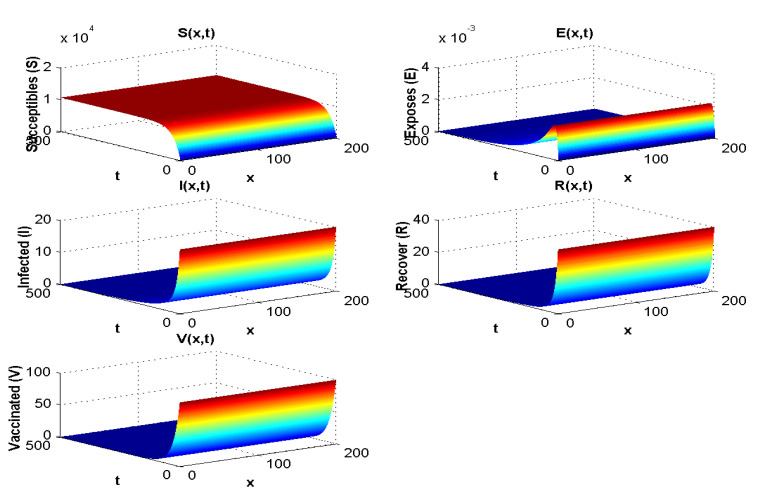
Effect of λ=0 on the spatial and temporal distribution when $R_{0}<1$.

In this section, all parameters used are the same as the ones used for experiment 1, except that in this case λ=0. The result of the simulation is presented in Figure 4.

#### 4.1.4. Experiment 4: Numerical Simulation When $R_{0}>1$ and λ=0

**Figure 5:**
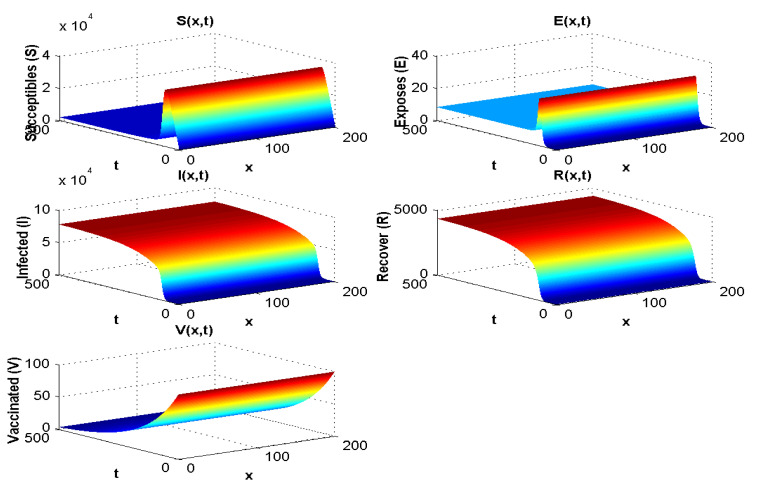
Effect of λ=0 on the spatial and temporal distribution when $R_{0}>1$.

In this section, all parameters used are the same as those used in experiment 2, except that in this case λ=0. The result of the simulation is presented in Figure 5.

### 4.2. Local Sensitivity of $R_{0}$

To know the effect of the parameters in system (1) on the control reproduction number $R_{0}$, we perform sensitivity analysis on $R_{0}$. This analysis is investigated analytically by computing $\frac{\partial R_{0}}{\partial Z}$, where $Z=(\sigma ,\Lambda ,\beta_{1},\beta ,d_{S},\lambda ,d_{V},\gamma ,d_{I},\theta ,d_{E}).$ The sensitivity of $R_{0}$ to each parameter is as follows:

$$\begin{array}{ccc} \frac{\partial R_{0}}{\partial \sigma } & = & \frac{\Lambda \left(\beta_{1}\left(d_{V}+\gamma \right)+\beta \lambda \right)}{\left(d_{S}+\lambda \right)\left(d_{V}+\gamma \right)\left(d_{I}+\theta \right)}\frac{d_{E}}{{\left(\sigma +d_{E}\right)}^{2}}>0\\ \frac{\partial R_{0}}{\partial \Lambda } & = & \frac{\sigma \left(\beta_{1}\left(d_{V}+\gamma \right)+\beta \lambda \right)}{\left(d_{S}+\lambda \right)\left(d_{V}+\gamma \right)\left(\sigma +d_{E}\right)\left(d_{I}+\theta \right)}>0\\ \frac{\partial R_{0}}{\partial \beta_{1}} & = & \frac{\sigma \Lambda (d_{V}+\gamma )}{\left(d_{S}+\lambda \right)\left(d_{V}+\gamma \right)\left(\sigma +d_{E}\right)\left(d_{I}+\theta \right)}>0\\ \frac{\partial R_{0}}{\partial \beta } & = & \frac{\sigma \Lambda \lambda }{\left(d_{S}+\lambda \right)\left(d_{V}+\gamma \right)\left(\sigma +d_{E}\right)\left(d_{I}+\theta \right)}>0\\ \frac{\partial R_{0}}{\partial d_{S}} & = & -\frac{\Lambda \left(\beta_{1}\left(d_{V}+\gamma \right)+\beta \lambda \right)}{\left(d_{V}+\gamma \right)\left(d_{I}+\theta \right)}\frac{1}{{\left(d_{S}+\lambda \right)}^{2}}<0\\ \frac{\partial R_{0}}{\partial \lambda } & = & \frac{\sigma \Lambda \left(\beta d_{S}-\beta_{1}\left(d_{V}+\gamma \right)\right)}{\left(d_{V}+\gamma \right)\left(\sigma +d_{E}\right)\left(d_{I}+\theta \right)}\\ \frac{\partial R_{0}}{\partial d_{V}} & = & -\frac{\sigma \Lambda }{\left(d_{S}+\lambda \right)\left(\sigma +d_{E}\right)\left(d_{I}+\theta \right)}\frac{\beta \lambda }{{\left(d_{V}+\gamma \right)}^{2}}<0 \end{array}$$

$$\begin{array}{ccc} \frac{\partial R_{0}}{\partial \gamma } & = & -\frac{\sigma \Lambda \left(\beta_{1}\left(d_{V}+\gamma \right)+\beta \lambda \right)}{\left(d_{S}+\lambda \right)\left(d_{I}+\theta \right)\left(\sigma +d_{E}\right)}\frac{1}{{\left(d_{V}+\gamma \right)}^{2}}<0\\ \frac{\partial R_{0}}{\partial d_{I}} & = & -\frac{\sigma \Lambda \left(\beta_{1}\left(d_{V}+\gamma \right)+\beta \lambda \right)}{\left(d_{S}+\lambda \right)\left(d_{V}+\gamma \right)\left(\sigma +d_{E}\right)}\frac{1}{{\left(d_{I}+\theta \right)}^{2}}<0\\ \frac{\partial R_{0}}{\partial \theta } & = & -\frac{\sigma \Lambda \left(\beta_{1}\left(d_{V}+\gamma \right)+\beta \lambda \right)}{\left(d_{S}+\lambda \right)\left(d_{V}+\gamma \right)\left(\sigma +d_{E}\right)}\frac{1}{{\left(d_{I}+\theta \right)}^{2}}<0\\ \frac{\partial R_{0}}{\partial d_{E}} & = & -\frac{\sigma \Lambda \left(\beta_{1}\left(d_{V}+\gamma \right)+\beta \lambda \right)}{\left(d_{S}+\lambda \right)\left(d_{V}+\gamma \right)\left(d_{I}+\theta \right)}\frac{1}{{\left(d_{E}+\sigma \right)}^{2}}<0. \end{array}$$

**Figure 6:**
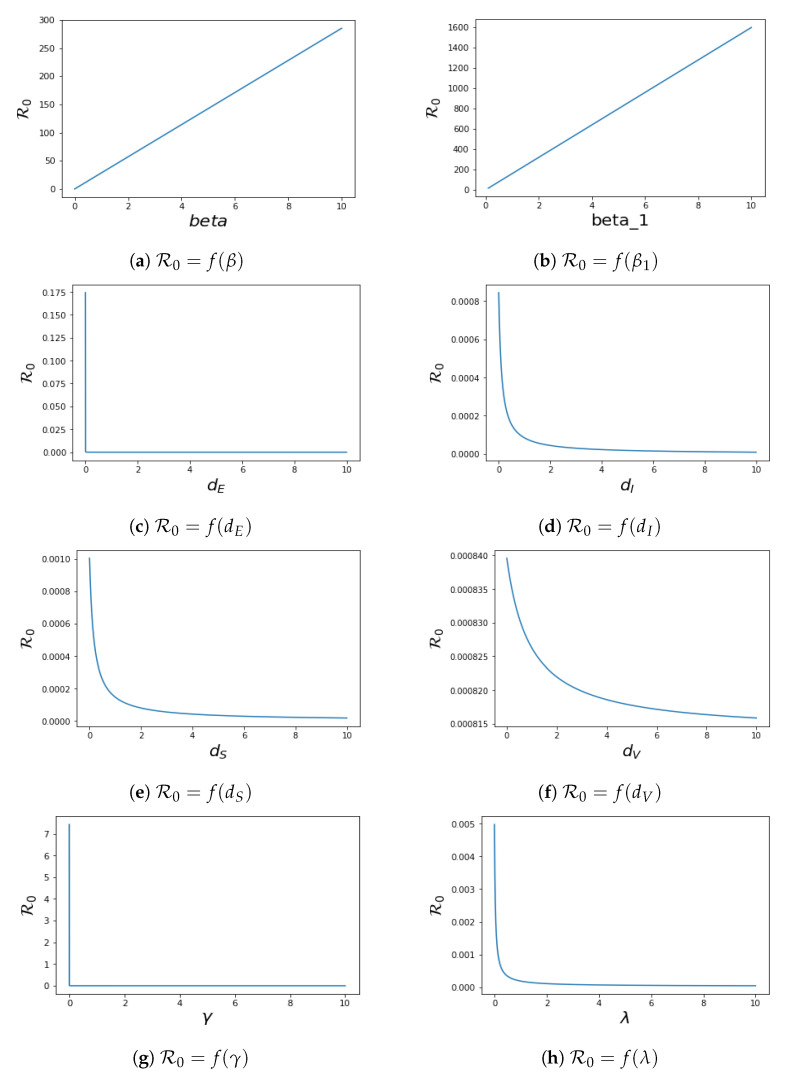

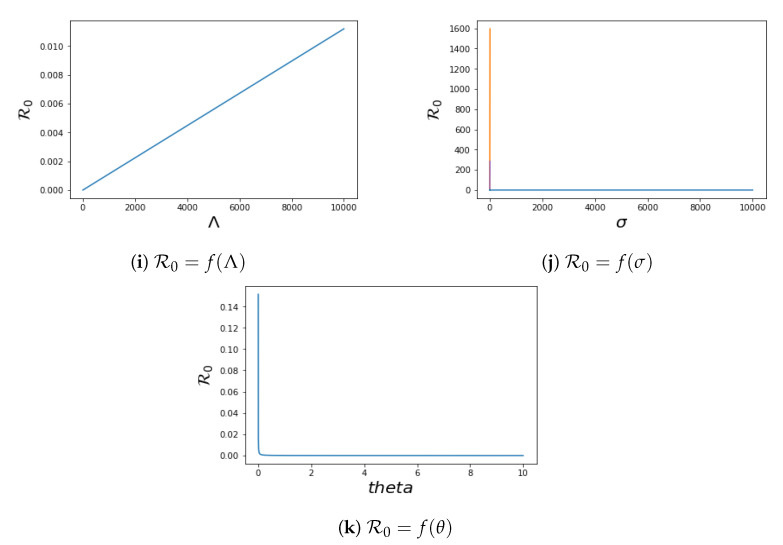
Sensitivity analysis of $R_{0}$ for the system (1).

We have the plot of $R_{0}$ in terms of their different parameters in Figure 6. The values of other parameters used for plotting while fixing the parameter that is plotted against $R_{0}$ for each plot are taken from Table 2.

The sensitivity index technique will help to measure the most sensitive parameters for the fundamental reproductive number $R_{0}$. The fundamental reproduction number’s normalised sensitivity index is provided by $S_{Z}^{R_{0}}=\frac{\partial R_{0}}{\partial Z}.\frac{Z}{R_{0}}$, where Z is a parameter, as defined earlier. We obtain

$$\begin{array}{cc} S_{\sigma }^{R_{0}} & =d_{E}/\left(d_{E}+\sigma \right),\\ S_{\Lambda }^{R_{0}} & =1,\\ S_{\beta_{1}}^{R_{0}} & =\frac{\beta_{1}\left(d_{V}+\gamma \right)}{\beta_{1}\left(d_{V}+\gamma \right)+\beta \lambda },\\ S_{\beta }^{R_{0}} & =\frac{\beta \lambda }{\beta_{1}\left(d_{V}+\gamma \right)+\beta \lambda },\\ S_{d_{S}}^{R_{0}} & =-d_{S}/\left(d_{S}+\lambda \right),\\ S_{\lambda }^{R_{0}} & =\frac{\lambda (-\beta_{1}\left(d_{V}+\gamma \right)-\beta \lambda +\beta \left(d_{S}+\lambda \right))}{\left(d_{S}+\lambda \right)\left(\beta_{1}\left(d_{V}+\gamma \right)+\beta \lambda \right)},\\ S_{d_{V}}^{R_{0}} & =-\frac{d_{V}\beta \lambda }{\left(d_{V}+\gamma \right)\left(\beta_{1}\left(d_{V}+\gamma \right)+\beta \lambda \right)},\\ S_{\gamma }^{R_{0}} & =-\frac{\gamma \beta \lambda }{\left(d_{V}+\gamma \right)\left(\beta_{1}\left(d_{V}+\gamma \right)+\beta \lambda \right)},\\ S_{d_{I}}^{R_{0}} & =-d_{I}/\left(d_{I}+\theta \right),\\ S_{\theta }^{R_{0}} & =-\theta /(d_{I}+\theta ),\\ S_{d_{E}}^{R_{0}} & =-d_{E}/\left(d_{E}+\sigma \right). \end{array}$$

The sensitivity indices obtained by using the parameters values in Table 2 are presented in Table 4. Four of the sensitivity indices are positive, while the others are negative, as can be seen in Table 4. We notice a difference in sensitivity between the parameters’ contact between *V* and *I* (β) and contact between *S* and *I* ($\beta_{1}$). This difference is minimal for β because the vaccinated population has developed some kind of immune system when in contact with the infected population, which is due to the fact that significant proportion of the population have been vaccinated, and maximal for $\beta_{1}$ because the parameter determines the spread of the disease during contact between the susceptible population and the infected population, hence the high sensitivity of this parameter. We conclude that increasing the recovery rate and vaccination rate will aid in decreasing the $R_{0}$, which affirms the effect of vaccination, and by extension, it is important to encourage a significant chunk of the population to get vaccinated, which will help to combat the spread of the virus.

## 5. Discussion

To demonstrate the influence of the spatial diffusion in the model, we used the method presented in [28] by using implicit–explicit finite differences, where the Laplacian term is discretised implicitly (for numerical stability, allowing for a relatively large time stepping), whereas nonlinear terms are handled explicitly. We assume Λ=0 and the diffusion term $d_{1}\Delta =d_{2}\Delta =0.00005$. Furthermore, we assume $S_{0}\left(x\right)=1.3+\cos\left(3\pi x\right)$ and $I_{0}=0.01\exp\left(-1000x\right)$ with $E_{0}=0,V_{0}=1,I_{0}=0$ and $I_{0}\left(1\right)=0.001$ in order to have a wave-like or exponential propagation of the disease. When varying β, we chose $\beta_{1}=1$ and λ=0.7 and when varying λ, we chose $\beta =\beta_{1}=1$. Other parameters were chosen from Table 2.

For the sensitivity analysis, we discovered that some of the parameters, i.e., $\Lambda ,\beta_{1},\beta ,$ and σ, cause $R_{0}$ to increase, while other parameters, corresponding to $d_{S},d_{E},\theta ,d_{I},d_{V}$, λ and γ, cause it to decrease.

When $R_{0}<1$, all compartments tend to zero except for the susceptible population, which increases with rate depending on Λ. Moreover, the extinction of other compartments is very fast (less than 50 days). For $R_{0}>1$, the susceptible and vaccinated populations tend towards zero, and the majority of the population becomes infected. Whenever $R_{0}<1$, the behaviour of the disease in the population does not change much with λ. However, when $R_{0}>1$, if we increase λ, the infected population will disappear, and the majority of the population becomes vaccinated. Hence, we cannot take λ<0 since the parameters are positive.

**Figure 7:**
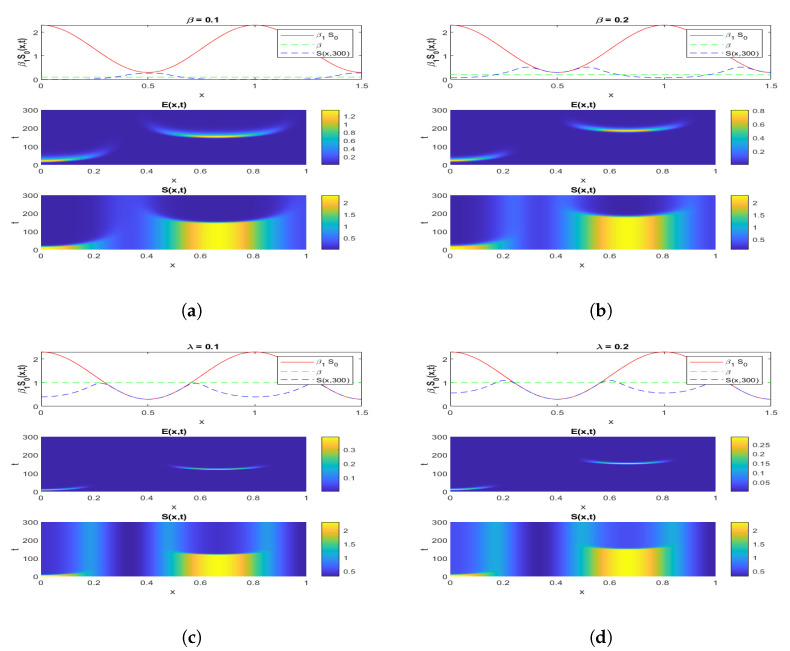
Simulation of an infection’s exponential phase propagating through a non-homogeneous population for the system (1), for different values of β and λ, as indicated.

Figure 3 shows both (i) the influence of a nonzero Λ flow of entrants into the susceptible population: the asymptotic value of S(x,∞) is not zero, contrary to what we observe in Figure 7, where Λ=0 and S(x,∞)=0, and (ii) the influence of the vaccination parameter λ, which, when it increases, increases the ratio V(x,∞)/S(x,∞) (it goes from the value 4.4 to the value 25 between Figure 2, where $\lambda =9.1\times 10^{-5}$ and Λ=350, and Figure 3, where λ=0.17 and Λ=15,000). When λ=0 (Figure 4 and Figure 5), we can see the influence of $R_{0}:S\left(x,\infty \right)=0$, if $R_{0}>1$, even if Λ is high, equal to 15,000 (Figure 5), and S(x,t) remains increasing with *t* if $R_{0}<1$, even if Λ is small, equal to 350 (Figure 4).

Figure 7a,b shows the influence of the parameter β and that of the diffusion: when β goes from 0.1 to 0.2, the asymptotic values S(x,∞)=0 and V(x,∞)=0 are reached later, and the initial shape of S(x,0) is kept longer. Figure 7c,d shows the influence of the parameter λ and that of the diffusion: when λ goes from 0.1 to 0.2, the asymptotic values S(x,∞)=0 and V(x,∞)=0 are also reached later, and the initial shape of S(x,0) is kept longer. If λ is small, that is, less than 0.5, the trend of S(x,300) will be close to linear. This shows, as expected, that we must wait a long time to obtain the asymptote for high values of β and λ, probably 500 or more time steps. Furthermore, for increasing λ, the disappearance of *S* would correspond to the appearance of a high asymptotic value of the evolution of *V*.

If the epidemic process starts with a non-homogeneous spatial initial condition for $\beta_{1}S_{0}\left(x,t\right)$, i.e., for the initial flow of exposed individuals resulting from a not spatially uniform contact rate per day between the susceptible and infected population, then Figure 7 shows the evolution of the susceptible and exposed sub-populations until their asymptotic state is reached. The susceptible individuals disappear progressively depending on both the exposition rate $\beta_{1}$ and the vaccination rate λ, while their number seems to remain constant for high values of λ.

## 6. Conclusions

In this paper, we have been able to develop a mathematical modelling of COVID-19 using diffusion equations built from some previous works to construct a more realistic model of COVID-19. Using this model, we were able to prove the existence, uniqueness, and local stability of constant stationary solutions. Another mathematical perspective we contributed was to build an appropriate Lyapunov function for these constant solutions in order to prove the global stability of the model. Then, we performed sensitivity analysis of $R_{0}$ in order to understand the dynamics of each parameter, which gives a better insight into how to control the evolution of the disease in the population, which is of interest to policy makers and public health experts. The numerical analysis showed agreement with the results of the qualitative analysis. Moreover, the proof of existence and stability of non-spatially homogeneous solutions and application of the model to time series data from different countries will be the subject of a future investigation. 

## Figures and Tables

**Table 1:** Parameters and their signification.

Parameters	Biological Signification of Parameters
Λ	Number of incoming susceptible per day
$d_{S}$	Mortality rate of susceptible per day
$\beta_{1}$	Contact between *S* and *I*
β	Contact between *V* and *I*
λ	Vaccination rate
$d_{E}$	Mortality rate of exposed population per day
σ	Infection rate
$d_{I}$	Mortality rate of infected population per day
$d_{R}$	Mortality rate of recovered population per day
θ	Recovery rate
$d_{V}$	Mortality rate of vaccinated population per day

**Table 2:** Values of the parameters when $R_{0}<1$.

Parameters	Values	References
Λ	350	Estimated
$d_{S}$	0.03324588	[26]
$\beta_{1}$	0.0000051	[26]
λ	0.000091	[26]
β	0.00001611	[26]
σ	17/100	[26]
$d_{E}$	0.003324588	[26]
$d_{I}$	0.06184588	[26]
θ	0.1109289	[26]
$d_{R}$	0.03324588	[26]
$d_{V}$	0.003324588	[26]
γ	0.15	Estimated

**Table 3:** Values of the parameters when $R_{0}>1$.

Parameters	Values	References
Λ	15,000	Estimated
$d_{S}$	0.003324588	[26]
$\beta_{1}$	0.0000051	[26]
λ	17/100	Estimated
β	0.00000091	[27]
σ	0.00001611	Estimated
$d_{E}$	0.003324588	[26]
$d_{I}$	0.06184588	[26]
θ	0.1109289	[26]
$d_{R}$	0.03324588	[26]
$d_{V}$	0.003324588	[26]
γ	0.95	[26]

**Table 4:** The sensitivity index of $R_{0}$ with respect to the parameters Z of the system (1).

Parameter	Sensitivity Index
σ	0.019
Λ	1
$\beta_{1}$	0.99
β	0.00187597
$d_{S}$	−0.99727
λ	−0.00085543
$d_{V}$	−0.00004068
γ	−0.00183557
$d_{I}$	−0.3579566
θ	−0.642
$d_{E}$	−0.019

## Data Availability

Data used for this research are available on public databases.
